# Myocardial Perfusion and Function Are Distinctly Altered by Sevoflurane Anesthesia in Diet-Induced Prediabetic Rats

**DOI:** 10.1155/2016/5205631

**Published:** 2015-12-28

**Authors:** Charissa E. van den Brom, Chantal A. Boly, Carolien S. E. Bulte, Rob F. P. van den Akker, Rick F. J. Kwekkeboom, Stephan A. Loer, Christa Boer, R. Arthur Bouwman

**Affiliations:** ^1^Department of Anesthesiology, VU University Medical Center, Boelelaan 1117, 1081 HV Amsterdam, Netherlands; ^2^Laboratory for Physiology, VU University Medical Center, Van der Boechorststraat 7, 1081 BT Amsterdam, Netherlands

## Abstract

Preservation of myocardial perfusion during surgery is particularly important in patients with increased risk for perioperative complications, such as diabetes. Volatile anesthetics, like sevoflurane, have cardiodepressive effects and may aggravate cardiovascular complications. We investigated the effect of sevoflurane on myocardial perfusion and function in prediabetic rats. Rats were fed a western diet (WD; *n* = 18) or control diet (CD; *n* = 18) for 8 weeks and underwent (contrast) echocardiography to determine perfusion and function during baseline and sevoflurane exposure. Myocardial perfusion was estimated based on the product of microvascular filling velocity and blood volume. WD-feeding resulted in a prediabetic phenotype characterized by obesity, hyperinsulinemia, hyperlipidemia, glucose intolerance, and hyperglycemia. At baseline, WD-feeding impaired myocardial perfusion and systolic function compared to CD-feeding. Exposure of healthy rats to sevoflurane increased the microvascular filling velocity without altering myocardial perfusion but impaired systolic function. In prediabetic rats, sevoflurane did also not affect myocardial perfusion; however, it further impaired systolic function. Diet-induced prediabetes is associated with impaired myocardial perfusion and function in rats. While sevoflurane further impaired systolic function, it did not affect myocardial perfusion in prediabetic rats. Our findings suggest that sevoflurane anesthesia leads to uncoupling of myocardial perfusion and function, irrespective of the metabolic state.

## 1. Introduction

Myocardial perfusion in relation to myocardial function determines the balance between myocardial energy supply and demand. During surgery, maintenance of myocardial oxygen balance is challenged. Extrinsic factors, like anesthetics and surgical stress, and intrinsic factors, such as cardiometabolic disease, affect myocardial oxygen supply and consumption. This altered balance may increase the vulnerability of the heart for an oxygen supply and demand mismatch and consequent ischemia [1, 2].

The volatile anesthetic sevoflurane exerts direct effects on the heart and circulation that jeopardise perioperative myocardial function and hemodynamic stability. Sevoflurane has vasodilating properties and is known to reduce coronary vascular resistance [3] and perfusion pressure [4, 5]. We showed that sevoflurane did not affect myocardial blood flow in cardiovascular healthy patients, while myocardial flow reserve was decreased [6]. Animal studies however showed that sevoflurane, when perfusion pressure remained constant, increased coronary blood flow in dogs [7] and decreased coronary flow reserve in isolated rat hearts [5]. In contrast, sevoflurane lowered blood pressure and decreased myocardial blood flow in healthy rats [8], dogs [9], and pigs [10].

While sevoflurane exerts contrasting effects on myocardial perfusion in healthy conditions, its vasodilatory impact may be more abundant in patients with cardiometabolic disease, like type 2 diabetes mellitus (T2DM). T2DM patients are more likely to develop coronary artery disease [11] and have an increased cardiovascular complication rate after major noncardiac surgery [12]. Because myocardial substrate metabolism and myocardial oxygen balance are altered in T2DM [13], the regulation of myocardial perfusion in these patients is particularly important during intraoperative circumstances, such as hypoperfusion. We previously found that myocardial perfusion, but not myocardial function, is preserved during hyperemia in glucose intolerant rats [14], while others showed myocardial perfusion defects in diabetic insulin resistant patients [15, 16] and T2DM patients during the postprandial state [17, 18]. The number of studies focusing on myocardial perfusion during sevoflurane anesthesia in subjects with cardiometabolic disease is however limited. We recently showed that sevoflurane decreased myocardial blood flow in T2DM patients and also a trend towards a lower vasodilator capacity was observed [19]. Taken together, these data suggest that the anesthesia-related alterations in myocardial perfusion and function may be more prominent in the presence of cardiometabolic disease.

Therefore, the purpose of the present study was to investigate the additional effect of sevoflurane anesthesia on myocardial perfusion and function in diet-induced prediabetic rats. We hypothesized that the impact of sevoflurane anesthesia is more abundant in the presence of cardiometabolic disease and thereby challenges perioperative regulation of myocardial perfusion and function.

## 2. Materials and Methods

### 2.1. Animals and Experimental Setup

This study was carried out in strict accordance with the European Convention for the Protection of Vertebrate Animals used for Experimental and Other Scientific Purposes. All experiments were approved by the Institutional Animal Care and Use Committee of the VU University (permit number ANES 12-04) and performed in compliance with the modern ARRIVE guidelines on animal research [20]. All surgeries were performed under S-Ketamine and diazepam anesthesia, and all efforts were made to minimize suffering.

The study was divided into two parts: (1) characterization of the phenotype induced by western diet feeding and (2) myocardial perfusion and function measurements during baseline conditions and sevoflurane exposure. The first part of the study was performed in a group of 16 male Wistar rats (baseline body weight: 264 ± 5 g; Charles River Laboratories, France), which were exposed to a western diet in combination with sucrose water (20%) (WD, *n* = 8) or control diet (CD, *n* = 8). After 8 weeks of diet exposure, rats underwent an oral glucose tolerance test. Rats were sacrificed after a 6 h fasting period by decapitation and trunk blood was collected for plasma determinations.

The second part of the study included 36 male Wistar rats (body weight 265 ± 7 g; Charles River Laboratories, France) that were exposed to either CD (*n* = 18) or WD (*n* = 18) as described above. After 8 weeks, rats underwent (contrast) echocardiography during baseline conditions and after 5 minutes of sevoflurane (2.0%) exposure.

All rats were housed in a temperature-controlled room (20–23°C; 40–60% humidity) under a 12/12 h light/dark cycle starting at 6.00 a.m. Body weight and caloric intake were determined on a weekly basis.

### 2.2. Diets

CD (Teklad 2016, Harlan, Horst, Netherlands) consisted of 20% kcal protein, 9% kcal fat, and 74% kcal carbohydrates (1804 kcal/kg starch, 200 kcal/kg sugars), whereas WD (D12451, Research Diets, New Brunswick, NJ) consisted of 20% kcal protein, 45% kcal fat, and 35% kcal carbohydrates (291 kcal/kg starch, 691 kcal/kg sugars) with 20% sucrose water (800 kcal/kg), totally containing 3300 kcal/kg and 4857 kcal/kg for CD and WD with sucrose water, respectively.

*Part 1*

### 2.3. Oral Glucose Tolerance Test

In the first part of the study, awake rats fasted overnight received an oral glucose load (2 g/kg of body weight). Blood glucose was measured from tail bleeds with a Precision Xceed Blood Glucose monitoring system (MediSense, UK) before (0) and 15, 30, 60, 90, and 120 min after glucose ingestion. At similar time points, plasma insulin (LINCO research, St. Charles, Missouri) levels were measured as described previously [14, 21].

### 2.4. Blood and Plasma Measurements

Plasma hematocrit levels were determined using microcentrifugation. Plasma glucose levels (Abcam, Cambridge, MA), plasma insulin (LINCO research, St. Charles, Missouri), plasma free fatty acids (WAKO NEFA-HR, Wako Pure Chemical Industries, Osaka, Japan), plasma triglyceride (Sigma, Saint Louis, Missouri), and plasma HDL and LDL/VLDL cholesterol (Abcam, Cambridge, MA) levels were measured from trunk blood as described previously [13, 14, 21, 22].

*Part 2*

### 2.5. Surgery

The rats in the second part of the study were anesthetized with 125 mg/kg S-Ketamine (Ketanest, Pfizer, Netherlands) and 4 mg/kg diazepam (Centrafarm, Netherlands) intraperitoneally. The trachea was intubated and lungs were mechanically ventilated (positive end-expiratory pressure, 1-2 cm H_2_O; respiratory rate, ~65 breaths/min; tidal volume, ~10 mL/kg) with oxygen-enriched air (40% O_2_/60% N_2_). Anesthesia was maintained by continuous infusion of 50 mg/kg/h S-Ketamine and 1.3 mg/kg/h diazepam intravenously via the tail vein. Respiratory rate was adjusted to maintain pH and partial pressure of carbon dioxide within physiological limits. Body temperature was maintained stable (36.7 ± 1.2°C) using a warm water underbody heating pad.

A catheter was placed in the right jugular vein for infusion of the contrast agent. The left carotid artery was cannulated for blood sampling, blood gas analyses (ABL50, radiometer, Copenhagen, Denmark), and measurements of arterial blood pressure (Safedraw Transducer Blood Sampling Set, Argon Medical Devices, Texas, USA). Arterial blood pressure, ECG, and heart rate were continuously recorded using PowerLab software (PowerLab 8/35, Chart 7.0; ADInstruments Pty, Ltd., Castle Hill, Australia). Mean arterial blood pressure was calculated according the following formula: 2/3 · diastolic  blood  pressure + 1/3 · systolic  blood  pressure. Rate pressure product (RPP) was calculated by the product of heart rate and systolic blood pressure and was used as an estimate of myocardial oxygen demand.

### 2.6. Preparation of Microbubbles

Microbubbles were prepared from perfluorobutane gas and stabilized with a monolayer of distearoyl phosphatidylcholine and PEG stearate. 1,2-Distearoyl-*sn*-glycero-3-phosphocholine (DSPC; Avanti Polar Lipids, Alabama, USA) and polyoxyethylene stearate (PEG40; Sigma, St. Louis, MO, USA) were dissolved in glycerol (10 mg/mL) and sonicated (Decon FS200, Decon Ultrasonics Ltd., Sussex, UK) at 40 kHz in an atmosphere of perfluorobutane (F2 Chemicals Ltd., Lancashire, UK) and vials were shaken in a Vialmix at 4500 rpm (Bristol-Myers Squibb Medical Imaging, Massachusetts, USA). As the gas was dispersed in the aqueous phase, microbubbles were formed, which were stabilized with a self-assembled lipid/surfactant monolayer. Freshly made bubbles were then washed twice to remove excessive DSPC and PEG40 and stored refrigerated in sealed vials in perfluorobutane atmosphere. A Multisizer 3 Coulter Counter (Beckman Coulter Inc., Miami, FL, USA) was used to measure the particle size distribution as well as the number of particles. The average bubble concentration was 1.58 · 10^9^ ± 0.36 · 10^9^ and the particle range was between 1 and 10 *μ*m. Microbubbles were diluted to a concentration of 200 · 10^6^ with degassed NaCl.

### 2.7. Myocardial Contrast Echocardiography

After surgery (contrast) echocardiography was performed to determine myocardial function and perfusion during baseline conditions and after 5 minutes of sevoflurane (2%) exposure. Contrast echocardiography was performed using a Siemens (ACUSON, Sequoia 512) equipped with a 14 MHz linear array transducer [13, 14]. Microbubbles were continuously infused into the jugular vein with a rate of 300 *μ*L/min using a dedicated syringe pump (Vueject, Bracco SA, Switzerland). After two minutes of microbubble infusion, perfusion images were taken from the long-axis view of the left ventricle.

Low acoustic power (mechanical index [MI] 0.20) was used for microbubble detection with a dynamic range of 50 dB. A perfusion sequence consisted of about 10 cardiac cycles of low MI imaging, followed by a burst of high acoustic power (MI 1.8) for complete contrast destruction. Subsequently, on average 20 cardiac cycles of low MI images were acquired to allow contrast replenishment in the myocardium. All data were stored for offline analysis.

### 2.8. Myocardial Contrast Echocardiography Analysis

Custom-designed software was used for analysis of the estimate of perfusion (Matlab, 7.10, R2010A, MathWorks Inc. Massachusetts, USA) [6, 14]. For each cardiac cycle, regions of interest were drawn in the end-systolic frame in the posterior wall in the long-axis view of the left ventricle. Myocardial signal intensities from the frames after microbubble destruction were corrected for background noise by subtracting the signal intensity of the first frame after microbubble destruction (*Y*_0_). These intensities were then fitted (*Y* = *Y*_0_ + (*A* − *Y*_0_)·(1 − exp^(−*β*·*x*)^)) for calculation of microvascular blood volume *A* and the microvascular filling velocity *β*, which corresponds to the capillary blood exchange rate. The estimate of perfusion was calculated as the product of *A* and *β* [23].

### 2.9. Echocardiography

Myocardial systolic function was determined with echocardiography [13, 14]. Briefly, left ventricular (LV) dimensions during end-systole (ES) and end-diastole (ED) were determined in the M- (motion-) mode of the parasternal short-axis view at the level of the papillary muscles. LV systolic function is represented by fractional shortening (FS) and fractional area change (FAC), which were calculated by the equations: FS = (EDD − ESD)/EDD · 100 and FAC = (EDD^2^ − ESD^2^)/EDD^2^ · 100. All parameters were averaged over at least three cardiac contractile cycles.

### 2.10. Statistical Analysis

Data were analyzed using Graphpad Prism 5.0 (La Jolla, USA) and presented as mean ± SD. Between group comparisons (CD versus WD) were performed using a Student *t*-test, whereas the effect of sevoflurane was tested with a two-way ANOVA with Bonferroni as post hoc test. *p* < 0.05 was considered as statistically significant.

## 3. Results

### 3.1. Western Diet Feeding Resulted in a Prediabetic Phenotype

**Figure 1:**
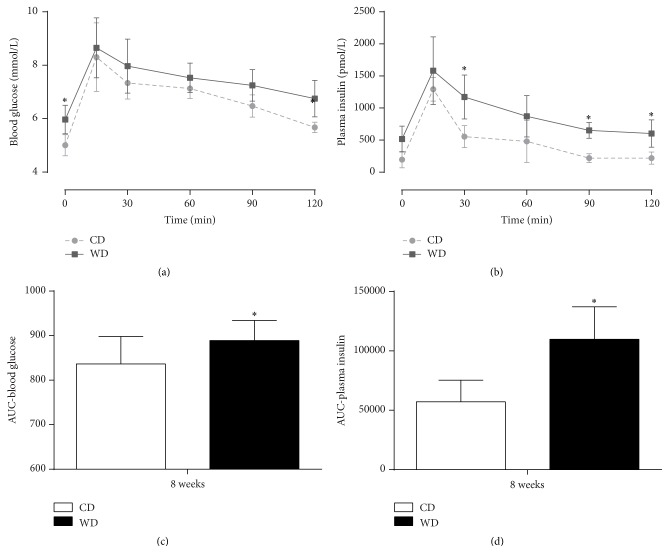
Oral glucose tolerance after 8 weeks of diet feeding. Blood glucose (a), plasma insulin (b), and area under the curve (AUC; (c) and (d)) during an oral glucose tolerance test in rats after 8 weeks of control diet (CD) and western diet (WD) feeding. Data are mean ± SD, *n* = 6, *t*-test, one- and two-way ANOVA with repeated measures, and Bonferroni post hoc analyses, ^*∗*^ *p* < 0.05 versus CD.

Eight weeks of western diet feeding resulted in a mild type 2 diabetic (prediabetic) phenotype with obesity, mild hyperglycemia, hyperinsulinemia, hyperlipidemia (Table 1), and glucose intolerance (Figure 1). Heart and liver weight and epididymal and perirenal fat pads were significantly increased in western diet-fed rats compared to controls. Furthermore, heart rate, systolic blood pressure, diastolic blood pressure, and mean arterial pressure remained unchanged (Table 1).

### 3.2. Impaired Myocardial Perfusion and Systolic Function in Prediabetic Rats

**Figure 2:**
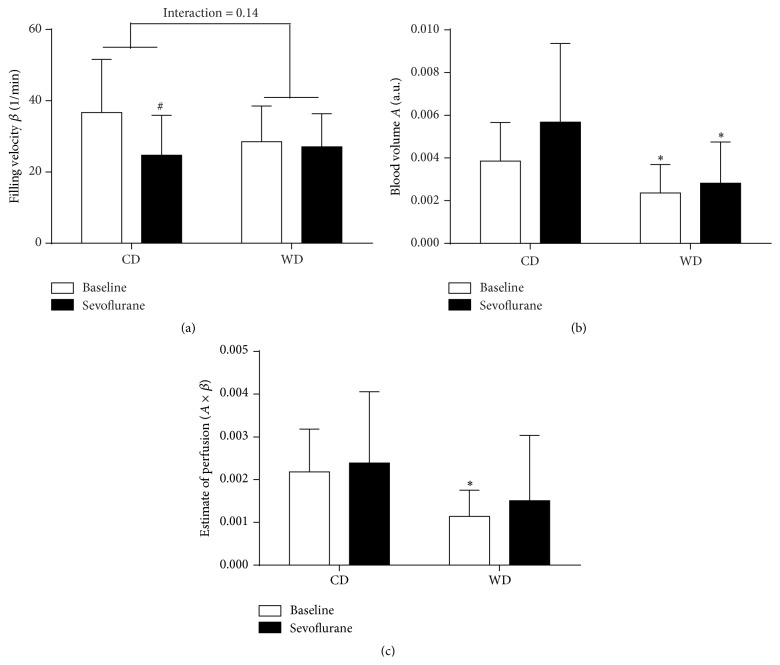
Effect of sevoflurane on myocardial perfusion in prediabetic rats. Microvascular blood volume *A* (a), microvascular filling velocity *β* (b), and estimate of perfusion (c) measured with contrast echocardiography in rats fed a control diet (CD) or western diet (WD) for 8 weeks during baseline conditions and after 5 minutes of sevoflurane exposure. Data are expressed as mean ± SD, *n* = 9–13; two-way ANOVA with Bonferroni post hoc analyses, ^*∗*^ *p* < 0.05 diet effect, ^#^ *p* < 0.05 sevoflurane effect.

Compared to healthy controls, western diet feeding tended to decrease microvascular filling velocity (*β*) and significantly decreased microvascular blood volume (*A*), which resulted in a significant reduction in the estimate of perfusion (Figure 2).

**Figure 3:**
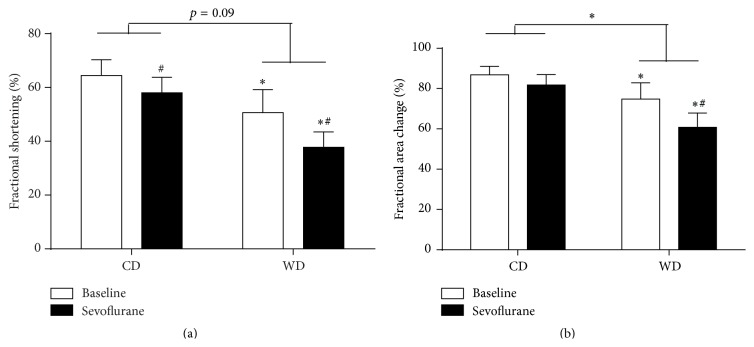
Effect of sevoflurane on systolic function in prediabetic rats. Systolic function, as represented by the fractional shortening (a) and fractional area change (b), measured with echocardiography in rats fed a control diet (CD) or western diet (WD) for 8 weeks during baseline conditions and after 5 minutes of sevoflurane exposure. Data are expressed as mean ± SD, *n* = 9–18; two-way ANOVA with Bonferroni post hoc analyses, ^*∗*^ *p* < 0.05 diet effect, ^#^ *p* < 0.05 sevoflurane effect.

Western diet feeding significantly increased end-systolic lumen diameter and diastolic wall thickness but did not affect end-diastolic lumen diameter and wall thickness during systole compared to control rats (Table 2). Fractional shortening and fractional area change were significantly decreased in western diet-fed rats compared to control animals, suggesting impaired systolic function (Figure 3).

### 3.3. Sevoflurane Further Impaired Systolic Function but Not Myocardial Perfusion in Prediabetic Rats

**Figure 4:**
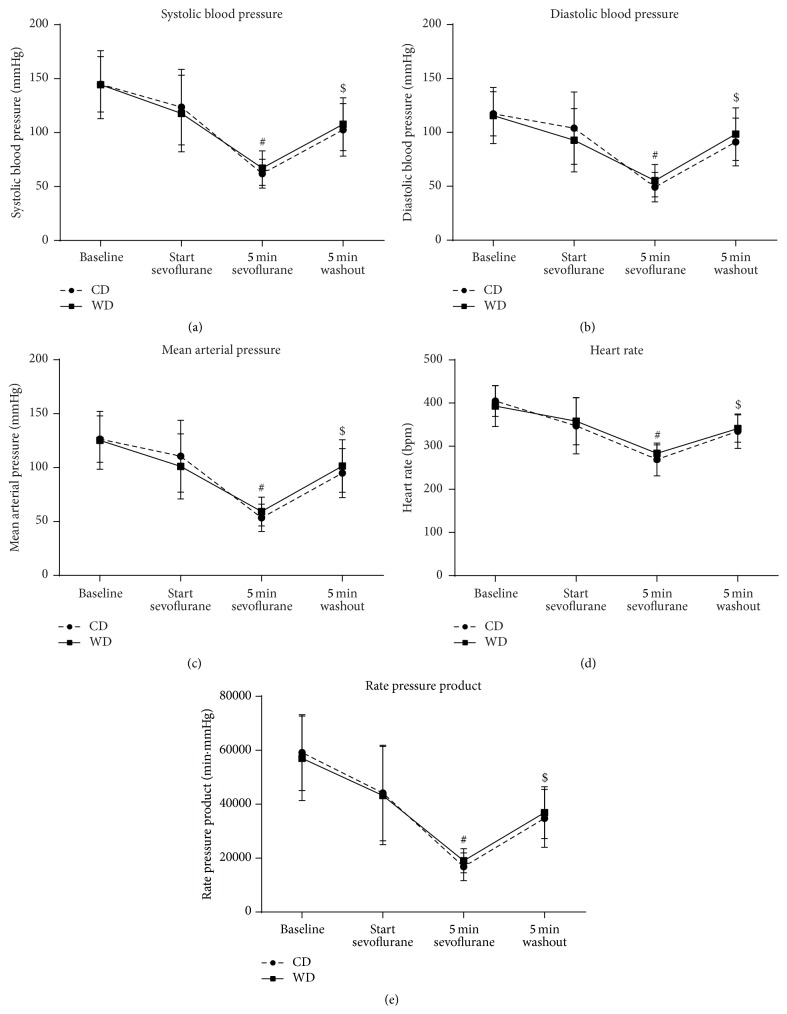
Hemodynamics during sevoflurane exposure. Systolic blood pressure (a), diastolic blood pressure (b), mean arterial pressure (c), heart rate (d), and rate pressure product (e) during baseline conditions, before sevoflurane exposure, after 5 minutes of sevoflurane and after 5 minutes of washout period in rats fed a control diet (CD) or western diet (WD) for 8 weeks. Data are mean ± SD, *n* = 16–18, two-way ANOVA with repeated measurements, and Bonferroni post hoc analyses, ^#^ *p* < 0.05 sevoflurane effect, ^$^ *p* < 0.05 washout effect.

Blood pressure, heart rate, and rate pressure product were significantly decreased after 5 minutes of sevoflurane exposure and significantly restored after a 5-minute washout period, without differences among diet groups (Figure 4).

Compared to baseline conditions, sevoflurane decreased the microvascular filling velocity (*β*) and tended to increase microvascular blood volume (*A*) in controls, while this observation was absent in western diet-fed animals. Overall, this resulted in an unchanged estimate of perfusion in both diet groups (Figure 2).

Sevoflurane additionally increased end-systolic lumen diameter in western diet-fed rats compared to baseline conditions, which resulted in further impaired systolic function in western diet-fed rats compared to control rats (Figure 3).

## 4. Discussion

In the present study, we examined the effect of sevoflurane anesthesia on myocardial perfusion and systolic function in western diet-fed rats. We found that short-term western diet feeding resulted in a mild type 2 diabetic phenotype (prediabetes), which was associated with impaired myocardial perfusion and systolic dysfunction. Sevoflurane had no additional effect on myocardial perfusion in healthy and prediabetic rats, while it impaired systolic function in healthy rats and even further impaired systolic function in prediabetic rats. These results suggest that sevoflurane leads to uncoupling of myocardial perfusion and function, irrespective of the metabolic state.

An interesting finding in this study is that myocardial perfusion and function were both decreased in diabetic rats compared to healthy controls. Previously, we found that myocardial perfusion and function were unaffected in high fat diet-induced glucose intolerant rats compared to healthy controls [14]. In the present study rats were exposed to a more severe western diet, which resulted in more pronounced disturbances in the cardiometabolic condition of the rats. Moreover, it was previously shown that the degree of reduction in myocardial blood flow reserve during acute hyperglycemia correlated to the severity of insulin resistance [24]. Taken together, these results suggest that impairment of myocardial perfusion is related to the severity of the diabetic state.

During surgery, the balance between myocardial energy supply and demand is challenged by extrinsic factors such as anesthetics. The volatile anesthetic sevoflurane exerts contrasting effects on myocardial perfusion in healthy animals and subjects. Previously it has been shown that sevoflurane did not alter myocardial blood flow in healthy rats [25] and cardiovascular healthy patients [6] compared to the awake condition. In contrast, others described decreased myocardial blood flow in healthy rats under general anesthesia with *α*-chloralose [8], dogs anesthetized with pentobarbital and fentanyl [9], and awake pigs [10]. The present study showed that sevoflurane did not affect myocardial perfusion, despite decreased arterial blood pressure, heart frequency, and rate pressure product in healthy rats. While myocardial perfusion remained unchanged, sevoflurane decreased microvascular filling velocity (*β*) and increased microvascular blood volume (*A*). The microvascular filling velocity is a parameter of the capillary exchange rate providing an estimate of the speed of erythrocytes through the capillaries, while microvascular blood volume suggests the surface area for exchange of nutrients and correlates with oxygen consumption. Our observations are in contrast with a previously performed study in cardiovascular healthy subjects by our group, where we showed that sevoflurane decreased myocardial blood volume and increased the microvascular filling velocity [6]. A possible mechanism to explain these differences may be derived by the differences in heart rate among species. We found a decrease in heart rate, while in healthy subjects an increase in heart rate was shown [6]. However, also decreased [8] or unchanged [25] heart rate in healthy rats is found during sevoflurane exposure. In addition to species variation and the use of different experimental techniques [23], administration of general anesthetics may explain the contrasting observations, as this may distinctly alter hemodynamics compared to the awake state. In the present study all rats were primarily sedated with S-ketamine and diazepam, because it is not feasible to perform contrast echocardiography in awake rats. As S-ketamine and diazepam also have intrinsic cardiodepressive effects [26, 27], this might have blurred the direct effect of sevoflurane on myocardial function. However, despite the use of several anesthetics, myocardial function was only slightly affected, whereas myocardial perfusion remained unaffected. Taken together, although sevoflurane anesthesia slightly impaired myocardial function, myocardial perfusion was not affected in healthy rats.

Preservation of myocardial perfusion during surgery is particularly important in patients with increased risk for perioperative cardiac complications, such as diabetes. Recently, our group showed that sevoflurane decreased myocardial perfusion in type 2 diabetic patients compared to healthy controls. Moreover, we observed a trend towards a lower endothelium-independent vasodilation capacity in type 2 diabetic patients under sevoflurane anesthesia, while endothelium-dependent vasodilation was not affected [19]. Our present results show that sevoflurane has a stronger cardiodepressive effect in prediabetic rats, whereas myocardial perfusion remained unaffected. Interestingly, systolic function was partly restored when sevoflurane was withdrawn (unpublished data). Under physiological conditions, myocardial blood flow and function are in balance [28]. In our prediabetic rats, myocardial perfusion and myocardial function were decreased. However, during sevoflurane anesthesia, myocardial perfusion was maintained, while myocardial function was further decreased in prediabetic rats. This uncoupling of perfusion and function suggests that, despite increased microvascular blood volume and decreased microvascular filling velocity, myocardial function cannot be maintained. Moreover, It should be kept in mind that the effects of sevoflurane are studied on top of S-Ketamine and diazepam anesthesia. However, the cardiodepressive effects of these agents do not explain our findings.

## 5. Conclusions

In conclusion, sevoflurane anesthesia maintained myocardial perfusion, while it impaired systolic function in healthy rats and even further impaired systolic function in prediabetic rats. Our findings suggest that sevoflurane anesthesia uncouples myocardial function and myocardial perfusion, irrespective of the metabolic state. This uncoupling might increase the vulnerability of the heart for an oxygen supply and demand mismatch and consequent ischemia during surgery.

## Figures and Tables

**Table 1:** Characteristics after 8 weeks of diet feeding.

	Control diet	Western diet
Caloric intake (kcal/100 gBW)	124 ± 6	129 ± 7
Blood/plasma characteristics (*n* = 8)		
6 h fasting plasma glucose (mmol/L)	8.6 ± 0.7	10.7 ± 1.1^*∗*^
6 h fasting plasma insulin (pmol/L)	933 ± 383	1524 ± 353^*∗*^
6 h fasting plasma free fatty acids (mmol/L)	0.26 ± 0.10	0.46 ± 0.24^*∗*^
6 h fasting plasma triglycerides (mmol/L)	0.68 ± 0.18	3.33 ± 1.19^*∗*^
6 h fasting plasma HDL cholesterol (mg/dL)	112.3 ± 11.8	61.9 ± 6.7^*∗*^
6 h fasting plasma LDL/VLDL cholesterol (mg/dL)	15.6 ± 2.2	27.3 ± 7.0^*∗*^
Hematocrit (%)	50.7 ± 3.0	47.9 ± 2.2^*∗*^
Body composition (*n* = 8)		
Body weight (g)	410 ± 27	478 ± 25^*∗*^
Heart weight (g)	1.19 ± 0.06	1.34 ± 0.15^*∗*^
Liver weight (g)	10.7 ± 1.0	14.2 ± 1.0^*∗*^
Epididymal fat weight (g)	6.0 ± 0.8	11.4 ± 2.1^*∗*^
Perirenal fat weight (g)	8.0 ± 1.5	16.7 ± 3.8^*∗*^
Tibia length (mm)	42.0 ± 0.5	41.8 ± 1.0
Hemodynamics (*n* = 18)		
Heart rate (bpm)	405 ± 36	393 ± 48
Systolic blood pressure (mmHg)	145 ± 26	144 ± 31
Diastolic blood pressure (mmHg)	117 ± 21	116 ± 26
Mean arterial pressure (mmHg)	126 ± 22	125 ± 27

Data are mean ± SD, *n* = 8–18; ^*∗*^ *p* < 0.05 versus control diet.

**Table 2:** Myocardial dimensions after 8 weeks of diet feeding during baseline and after sevoflurane exposure.

	Baseline		Sevoflurane	
	Control diet	Western diet	Control diet	Western diet
Diastolic lumen diameter (mm)	5.7 ± 0.6	5.5 ± 0.8	5.4 ± 0.8	5.5 ± 0.8
Systolic lumen diameter (mm)	2.0 ± 0.4	2.7 ± 0.7^*∗*^	2.3 ± 0.5	3.4 ± 0.6^*∗*#^
Diastolic wall thickness (mm)	1.8 ± 0.1	1.9 ± 0.2^*∗*^	1.6 ± 0.2	1.9 ± 0.2^*∗*^
Systolic wall thickness (mm)	3.3 ± 0.3	3.1 ± 0.4	3.0 ± 0.4	2.9 ± 0.2

Data are mean ± SD, *n* = 9–18; two-way ANOVA with Bonferroni post hoc analyses, ^*∗*^ *p* < 0.05 diet effect, ^#^ *p* < 0.05 sevoflurane effect.

## References

[1] Dole W. P. (1987). Autoregulation of the coronary circulation. *Progress in Cardiovascular Diseases*.

[2] Hoffman J. I. E., Spaan J. A. E. (1990). Pressure-flow relations in coronary circulation. *Physiological Reviews*.

[3] Malan T. P., DiNardo J. A., Isner R. J. (1995). Cardiovascular effects of sevoflurane compared with those of isoflurane in volunteers. *Anesthesiology*.

[4] Park K. W. (2002). Cardiovascular effects of inhalational anesthetics. *International Anesthesiology Clinics*.

[5] Larach D. R., Schuler H. G. (1991). Direct vasodilation by sevoflurane, isoflurane, and halothane alters coronary flow reserve in the isolated rat heart. *Anesthesiology*.

[6] Bulte C. S. E., Slikkerveer J., Kamp O. (2013). General anesthesia with sevoflurane decreases myocardial blood volume and hyperemic blood flow in healthy humans. *Anesthesia and Analgesia*.

[7] Crystal G. J., Zhou X., Gurevicius J. (2000). Direct coronary vasomotor effects of sevoflurane and desflurane in in situ canine hearts. *Anesthesiology*.

[8] Conzen P. F., Vollmar B., Habazettl H., Frink E. J., Peter K., Messmer K. (1992). Systemic and regional hemodynamics of isoflurane and sevoflurane in rats. *Anesthesia and Analgesia*.

[9] Hirano M., Fujigaki T., Shibata O., Sumikawa K. (1995). A comparison of coronary hemodynamics during isoflurane and sevoflurane anesthesia in dogs. *Anesthesia and Analgesia*.

[10] Manohar M., Parks C. M. (1984). Porcine systemic and regional organ blood flow during 1.0 and 1.5 minimum alveolar concentrations of sevoflurane anesthesia without and with 50% nitrous oxide. *Journal of Pharmacology and Experimental Therapeutics*.

[11] Preis S. R., Pencina M. J., Hwang S.-J. (2009). Trends in cardiovascular disease risk factors in individuals with and without diabetes mellitus in the Framingham Heart Study. *Circulation*.

[12] Lee T. H., Marcantonio E. R., Mangione C. M. (1999). Derivation and prospective validation of a simple index for prediction of cardiac risk of major noncardiac surgery. *Circulation*.

[13] van den Brom C. E., Huisman M. C., Vlasblom R. (2009). Altered myocardial substrate metabolism is associated with myocardial dysfunction in early diabetic cardiomyopathy in rats: studies using positron emission tomography. *Cardiovascular Diabetology*.

[14] van den Brom C. E., Bulte C. S. E., Kloeze B. M., Loer S. A., Boer C., Bouwman R. A. (2012). High fat diet-induced glucose intolerance impairs myocardial function, but not myocardial perfusion during hyperaemia: a pilot study. *Cardiovascular Diabetology*.

[15] Nasr G., Sliem H. (2010). Silent myocardial ischemia in prediabetics in relation to insulin resistance. *Journal of Cardiovascular Disease Research*.

[16] Nasr G., Sliem H. (2011). Silent ischemia in relation to insulin resistance in normotensive prediabetic adults: early detection by single photon emission computed tomography (SPECT). *International Journal of Cardiovascular Imaging*.

[17] Scognamiglio R., Negut C., De Kreutzenberg S. V., Tiengo A., Avogaro A. (2005). Postprandial myocardial perfusion in healthy subjects and in type 2 diabetic patients. *Circulation*.

[18] Scognamiglio R., Negut C., De Kreutzenberg S. V., Tiengo A., Avogaro A. (2006). Effects of different insulin regimes on postprandial myocardial perfusion defects in type 2 diabetic patients. *Diabetes Care*.

[19] Bulte C. S. E., van den Brom C. E., Loer S. A., Boer C., Bouwman R. A. (2014). Myocardial blood flow under general anaesthesia with sevoflurane in type 2 diabetic patients: a pilot study. *Cardiovascular Diabetology*.

[20] Kilkenny C., Browne W. J., Cuthill I. C., Emerson M., Altman D. G. (2010). Improving bioscience research reporting: the ARRIVE guidelines for reporting animal research. *PLoS Biology*.

[21] Ouwens D. M., Diamant M., Fodor M. (2007). Cardiac contractile dysfunction in insulin-resistant rats fed a high-fat diet is associated with elevated CD36-mediated fatty acid uptake and esterification. *Diabetologia*.

[22] van den Brom C. E., Bosmans J. W. A. M., Vlasblom R. (2010). Diabetic cardiomyopathy in Zucker diabetic fatty rats: the forgotten right ventricle. *Cardiovascular Diabetology*.

[23] Bulte C. S. E., Slikkerveer J., Meijer R. I. (2012). Contrast-enhanced ultrasound for myocardial perfusion imaging. *Anesthesia and Analgesia*.

[24] Abdelmoneim S. S., Hagen M. E., Mendrick E. (2013). Acute hyperglycemia reduces myocardial blood flow reserve and the magnitude of reduction is associated with insulin resistance: a study in nondiabetic humans using contrast echocardiography. *Heart and Vessels*.

[25] Crawford M. W., Lerman J., Saldivia V., Carmichael F. J. (1992). Hemodynamic and organ blood flow responses to halothane and sevoflurane anesthesia during spontaneous ventilation. *Anesthesia and Analgesia*.

[26] Plante E., Lachance D., Roussel É., Drolet M.-C., Arsenault M., Couet J. (2006). Impact of anesthesia on echocardiographic evaluation of systolic and diastolic function in rats. *Journal of the American Society of Echocardiography*.

[27] Stein A. B., Tiwari S., Thomas P. (2007). Effects of anesthesia on echocardiographic assessment of left ventricular structure and function in rats. *Basic Research in Cardiology*.

[28] Heusch G., Schulz R. (1999). The relation of contractile function to myocardial perfusion. Perfusion-contraction match and mismatch. *Herz*.

